# Antispasmodic and Antidiarrheal Activities of *Valeriana hardwickii* Wall. Rhizome Are Putatively Mediated through Calcium Channel Blockade

**DOI:** 10.1155/2011/304960

**Published:** 2011-03-03

**Authors:** Samra Bashir, Raafia Memon, Anwar H. Gilani

**Affiliations:** ^1^Department of Biological and Biomedical Sciences, Aga Khan University Medical College, Karachi 74800, Pakistan; ^2^Department of Pharmacy, Bahauddin Zakariya University, Multan, Pakistan

## Abstract

*Valeriana hardwickii* is indigenous to Pakistan, Burma and Ceylon, where it is traditionally being used as an antispasmodic and antidiarrheal, besides its culinary use as spice. The aim of this paper was to provide pharmacological validation to these medicinal uses. The crude aqueous-methanolic extract of *Valeriana hardwickii* rhizome (Vh.Cr) was studied on isolated rabbit jejunum and castor oil-induced diarrhea in mice for spasmolytic and antidiarrheal properties, respectively. Vh.Cr caused concentration-dependent (0.01–1 mg/mL) relaxation of spontaneous contractions in isolated rabbit jejunum and inhibited K^+^-induced contractions (0.01–0.3 mg/mL), similar to verapamil, suggestive of calcium channel blockade (CCB). The CCB effect was confirmed when pretreatment of the jejunum preparations with Vh.Cr produced a concentration-dependent (0.03–0.1 mg/mL) rightward shift in the Ca^++^ concentration-response curves, as caused by verapamil. Vh.Cr exhibited dose-dependent (100–300 mg/kg) protection against castor oil-induced diarrhea in mice. Loperamide, a standard antidiarrheal drug, similarly prevented the diarrhea. These data indicate the presence of CCB effect in the extract of *Valeriana hardwickii* rhizome, possibly mediating its antispasmodic and antidiarrheal activities and provide a scientific base for its traditional use in hyperactive gut disorders.

## 1. Introduction

Gastrointestinal diseases particularly constipation and diarrhea are affecting 70% of the population worldwide [1]. Diarrhea is one of the leading causes of mortality in developing countries and prevails as an ailment underlying 1.5–2 million deaths among children under 5 years of age [2]. Medicinal plants are usually preferred to treat gastrointestinal disorders, for example, constipation and diarrhea, because they contain multiple constituents with effect-enhancing and/or side effect-neutralizing potential [3], and, hence are considered relatively safe in prolonged use. The seed husk of *Plantago ovata* (psyllium husk) is a typical example of a plant-based remedy equally popular among traditional healers and modern physicians as a complementary and alternative therapy [4]. There has been increased global interest in traditional medicine and there are efforts underway to monitor and regulate herbal drugs and traditional medicine [5]. 

*Valeriana hardwickii *Wall. (family; Valerianaceae), commonly known as valerian and locally as bal-Charr, is a wild-growing herb, native to Pakistan, Burma and Ceylon. Valerian is indigenously used as a spice as well as medicine [6]. It is considered useful as a diaphoretic, antiperiodic, stimulant, cephalic tonic, antiepileptic, antihelmenthic, sedative, diuretic, aphrodisiac, emmenagogue, deobstruent, spasmolytic and antidiarrheal. The vertical rhizome and attached roots of valerian are the parts used medicinally [7]. 

Phytochemical investigations have shown the *Valeriana hardwickii* rhizome to possess essential oil [6] constituting *α*-kessyl acetate, valeracetate, 8-epikessyl glycol diacetate, maaliol, kessanyl acetate [8], bornyl acetate, methyl linoleate, cuparene, *α*-cedrene [9] and epoxysesquithujene [10] among terpenoids.

There is a scarcity of pharmacological data on *Valeriana hardwickii* and its constituents. In this study, we report the presence of spasmolytic and antidiarrheal activities in *Valeriana hardwickii*, mediated possibly through calcium antagonistic mechanism, which may explain the traditional use of the plant in abdominal cramps and diarrhea.

## 2. Material and Methods

### 2.1. Drugs and Animals

The reference drugs, acetylcholine perchlorate, loperamide, potassium chloride and verapamil hydrochloride were purchased from Sigma Chemicals Co., St. Louis, MO, U. S. A. All other chemicals used were of the available analytical grade. All drugs were dissolved in distilled water and dilutions were made fresh in normal saline (0.9% sodium chloride) on the day of experiment.

Balb-C mice (20–25 g) and rabbits (1.5–2.0 kg) of local breed, of either sex, were used for this study. Animals were housed at the Animal House of the Aga Khan University, maintained at 23–25°C and were given a standard diet and tap water *ad libitum*. Animals had free access to water but food was withdrawn 24 hours prior to experiments. Experiments performed complied with the rulings of the Institute of Laboratory Animal Resources, Commission on Life Sciences, National Research Council [11]. All procedures and experimental protocols were approved by the Ethical Committee for Research on Animals (ECRA) at the Aga Khan University.

### 2.2. Plant Material and Preparation of the Crude Extract

*Valeriana hardwickii* rhizomes were purchased from an authentic herb supplier in the local market of Karachi, Pakistan. A sample of the plant material was submitted to the herbarium of Natural Products Research Unit at the Department of Biological and Biomedical Sciences of the Aga Khan University, with a voucher number VH-RT-09-06-67b. The dried rhizome was made free of dirt and ground to powder, using commercial mill. The ground rhizome was soaked in 70% methanol in water (v/v) at room temperature for three days with occasional shaking [12]. After filtering through a single layer of muslin cloth, the final filtrate was collected by passing it through a Whatman grade 1 filter paper. This procedure of soaking the rhizome residue and filtration was repeated twice. All the filtrates were combined and evaporated to dryness on a rotary evaporator under reduced pressure to a thick and dark-brown material, the crude extract of *Valeriana hardwickii* rhizome (Vh.Cr), the yield was 15%.

The normal saline, used for solubilization of the extract, had no effect on tissue contractility in the control experiments.

### 2.3. Isolated Rabbit Jejunum

The spasmolytic activity of the plant material was studied by using isolated rabbit jejunum as described previously [13, 14]. Intestinal segments of 2 cm length were suspended in a 10 mL tissue bath containing Tyrode's solution, bubbled with 95% oxygen in carbon dioxide and maintained at 37°C. Composition of the Tyrode's solution was KCl 2.68, NaCl 136.9, MgCl_2_ 1.05, NaHCO_3_ 11.90, NaH_2_PO_4_ 0.42, CaCl_2_ 1.8, and glucose 5.55 mM. Intestinal isotonic responses were recorded via force transducer coupled to a Transbridge (model TBM4M, World Precision Instruments, Hertfordshire, UK) and PowerLab data acquisition system (model ML845, ADInstruments, Sydney, Australia). Each tissue was allowed to equilibrate for at least 30 min before the addition of any drug. Under these experimental conditions, the rabbit jejunum exhibits spontaneous rhythmic contractions, allowing testing the relaxant (spasmolytic) activity without the use of an agonist [13, 15]. 

To assess whether the spasmolytic activity of the test substances was through calcium channel blockade, Potassium was used to depolarize the preparations as described by Farre et al. [16]. High potassium (80 mM) was added to the tissue bath, which produced a sustained contraction. Test material was then added in a cumulative fashion to obtain concentration-dependent inhibitory responses [17]. The relaxation of intestinal preparations, precontracted with potassium (80 mM), was expressed as a percent of the control response mediated by potassium. Contraction of smooth muscle induced by potassium is known to be mediated via influx of calcium from extracellular fluid and the substance, which inhibits this contraction, is considered to act through blockade of calcium channels [18]. 

To confirm the calcium antagonist activity of test substances, the tissue was allowed to stabilize in normal Tyrode's solution, which was then replaced with calcium-free Tyrode's solution containing EGTA (0.1 mM) for 30 min in order to remove calcium from the tissues. This solution was further replaced with potassium-rich and calcium-free Tyrode's solution, having the following composition: KCl 50, NaCl 91.04, MgCl_2_ 1.05, NaHCO_3_ 11.90, NaH_2_PO_4_ 0.42, glucose 5.55, and EGTA 0.1 mM. Following an incubation period of 30 min, control concentration-response curves (CRCs) of calcium were obtained. When the control CRCs of calcium were found superimposable (usually after two cycles), the tissue was pretreated with the plant extract for 60 min to test the possible calcium channel blocking effect. The CRCs of calcium were reconstructed in the presence of different concentrations of Vh.Cr.

### 2.4. Antidiarrheal Activity

Antidiarrheal effect of Vh.Cr was tested on castor oil-induced diarrhea in mice by slight modification of the method previously used by Wang et al. [19] in terms of calculation of castor oil dose per kg of body weight basis instead of using fixed dose for each animal. Twenty-five mice, fasted for 24 hr before the experiment, were divided into five groups of five mice each. The animals were housed in individual cages. The first group, served as vehicle control, received saline (10 mL/kg, orally) without subsequent castor oil treatment. The second group received saline followed by castor oil treatment and thus acted as negative control. The third group of mice was treated with loperamide (10 mg/kg), as positive control, whereas, the other two groups received Vh.Cr, 100 and 300 mg/mL, respectively. One hr after the treatment, each animal received 10 mL/kg of castor oil orally through a feeding needle. After 4 hours, the cages were inspected for the presence of the typical diarrheal droppings; their absence was noted as a positive result, indicating protection from diarrhea at that time.

### 2.5. Statistical Analysis

All the data expressed are mean ± standard error of the mean (SEM) and the median effective concentrations (EC_50_ values) with 95% confidence intervals (CI).The statistical parameter applied in the castor oil-induced diarrhea test is chi-square test. *P* < .05 was noted as significantly different. Concentration-response curves (CRCs) were analyzed by nonlinear regression using GraphPad program (GraphPad, San Diego, CA, USA).

## 3. Results

### 3.1. Effect on Rabbit Jejunum

**Figure 1:**
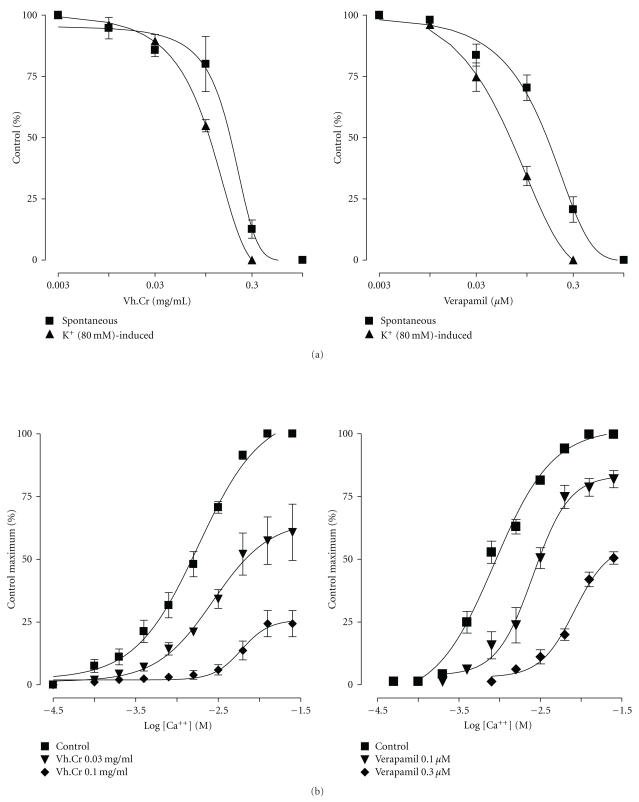
(a) Shows concentration-dependent inhibitory effects of the crude extract of *Valeriana hardwickii *(Vh.Cr) and verapamil on spontaneous and potassium-induced contractions, and (b) shows concentration-response curves of Ca^++^ in the absence and presence of increasing concentrations of Vh.Cr and verapamil in isolated rabbit jejunum preparations. Symbols represent mean ± S.E.M., *n* = 4-5.

Vh.Cr caused a concentration-dependent inhibition of spontaneous and potassium-induced contractions of isolated jejunum preparations with respective EC_50_ value of 0.175 mg/mL (0.14–0.23) and 0.11 mg/mL (0.1-0.2). Similarly, verapamil relaxed both types of contractions with EC_50_ values of 0.16 *μ*M (0.13–0.21) and 0.07 *μ*M (0.05–0.08), respectively (Figure 1(a)). At the tested concentration ranges, Vh.Cr was equally effective but significantly (*P* < .01) less potent than verapamil in both of the aforementioned activities. Vh.Cr also caused a concentration-dependent (0.03 and 0.1 mg/mL) rightward shift in the calcium concentration-response curves (Figure 1(b)), similar to verapamil (0.1 and 0.3 *μ*M).

### 3.2. Effect on Castor Oil-Induced Diarrhea

**Figure 2:**
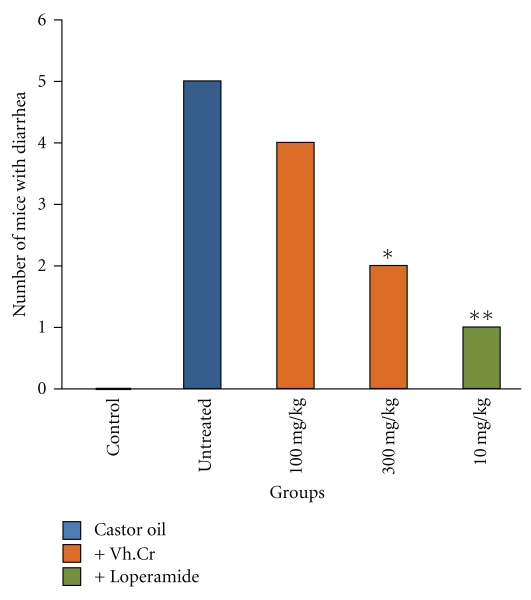
Bar-chart showing the protective effect of *Valeriana hardwickii* rhizome extract (Vh.Cr) and loperamide on castor oil-induced diarrhea in mice. *P < .05, and **P < .01 versus untreated group.

Vh.Cr exhibited a dose-dependent (100 and 300 mg/kg) antidiarrheal effect against castor oil-induced diarrhea in mice (Table 1 and Figure 2). The vehicle control group (saline treated only) did not show diarrhea in any of the animals, while all mice in the negative control group showed diarrhea. In animals pretreated with Vh.Cr, the extract showed 20% protection against diarrhea at a dose of 100 mg/kg and 60% protection at 300 mg/kg (*P* < .05 versus negative control). Loperamide (10 mg/kg) showed 80% protection from diarrhea in the positive control group. At the tested doses, Vh.Cr was significantly (*P* < .01) less effective than loperamide.

## 4. Discussion

The aqueous-methanolic extract of *Valeriana hardwickii* rhizome caused inhibition when tested on spontaneously contracting rabbit jejunum preparations, thus showing spasmolytic action. The contraction of smooth muscle preparations, including rabbit jejunum, is dependent upon an increase in the cytoplasmic free calcium, which activates the contractile elements [20]. The increase in intracellular calcium is due to either influx via voltage dependant calcium channels (VDCCs) or to release from intracellular stores in the sarcoplasmic reticulum. Periodic depolarization regulates the spontaneous movements of intestine and at the height of depolarization the action potential appears as a rapid influx of calcium via VDCCs [21]. The inhibitory effect of the plant extract on spontaneous movements of rabbit jejunum may be due to interference either with the calcium release or with the calcium influx through VDCCs. 

**Figure 3:**
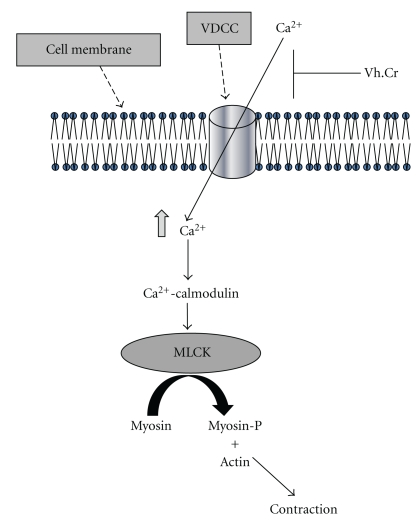
Schematic diagram of proposed mechanism of *Valeriana hardwickii* crude extract (Vh.Cr)-induced inhibitory effect on intestinal muscle contraction causing its spasmolytic action. VDCC: Voltage dependent calcium channels; MLCK: myosin light chain kinase; Myosin-P: phosphorylated myosin.

In our earlier studies, we have observed that the spasmolytic effect of the medicinal plants is usually mediated through calcium channel blockade [13, 15]. To see whether the spasmolytic effect of this plant is also mediated via the same mechanism, the crude extract was tested on high potassium-induced (80 mM) sustained contraction. The valerian extract caused relaxation of the contraction, similar to verapamil, a standard calcium channel blocker [22]. Potassium at high doses (**>**30 mM) is known to cause smooth muscle contractions through opening of VDCs, thus allowing influx of extracellular calcium causing a contractile effect and a substance causing inhibition of high potassium-induced contraction is considered a blocker of calcium influx [18, 23]. The presence of calcium antagonist constituent(s) was confirmed when the plant extract caused rightward shift in the calcium-concentration-response curves, constructed in the applied potassium-rich and calcium-free medium, similar to that caused by verapamil. Calcium antagonists, such as verapamil form an important therapeutic group, which is particularly employed in the treatment of different cardiovascular diseases [24]. The common characteristic of these drugs is their concentration-dependent inhibition of the slow entry of calcium and their capacity for reversal of this effect by calcium [22]. The observed effect of the plant extract to inhibit the induced contractions, followed by displacing the calcium curves similar to that by verapamil strongly suggests the presence of a calcium antagonist(s) in the plant extract mediating its effect through inhibition of calcium influx via VDCCs (Figure 3). 

The antidiarrheal property of *Valeriana hardwickii* was determined by its protective effect against the castor oil-induced diarrhea in mice. The induction of diarrhea with castor oil results from its hydrolytic product, ricinoleic acid [25], which produces changes in the transport of electrolytes and water resulting in the generation of giant contractions of the transverse and distal colon [26]. Thus, a potential antidiarrheal agent may exhibit its antidiarrheal effect by inhibiting bowel contractions. Vh.Cr exhibited a dose-dependent protective effect against diarrhea, which is in accordance with the expectation, as calcium antagonists are reported to possess antidiarrheal property [27]. 

Medicinal and aromatic plants have played an important role in sociocultural, spiritual, and health care needs of rural and tribal people of the emerging and developing countries [28]. According to an estimate, over 80% of the developing world's population still rely on the traditional medicines (mainly herbs) to cater to their health care needs despite advances in modern medicine, which is mainly attributed to strong cultural believes, accessibility, and affordability [29, 30]. The same is true for Pakistan, where there are at least 45,000 traditional healers of whom about three-quarters are practicing in rural and tribal areas and well over 70% of the country's population attend their clinics [31]. Despite such a high dependency, scientific investigations on traditional remedies have not been widely carried out. This gap between the use of traditional remedies and their scientific basis exists mainly due to the lack of active interactions between traditional and modern health professionals. 

Pharmacopoeias of several countries describe a large number of plant species being used as remedies for abdominal spasm and diarrhea. Pharmacological studies on some of these plants have revealed them to act through one or a combination of multiple mechanisms, mainly anticholinergic, calcium channel, blockade, opening of potassium channels and phosphodiesterase inhibition [13, 32, 33]. 

Results of this study conducted on *Valeriana hardwickii *clearly indicate the presence of both spasmolytic and antidiarrheal effects in its rhizomes, mediating putatively through calcium channel blockade, and this study provides sound mechanistic basis for the use of plant in gastrointestinal disorders, such as abdominal cramps and diarrhea.

## Figures and Tables

**Table 1:** Effect of *Valeriana hardwickii* rhizome extract (Vh.Cr) on castor oil-induced diarrhea in mice.

Treatment (oral)	No. of mice/5 with diarrhea	% protection
Vehicle control (Saline 10 mL/kg)	0	—
Castor oil (10 mL/kg)	5	0
+ Vh.Cr (100 mg/kg)	4	20
+ Vh.Cr (300 mg/kg)	2*	60
+ Loperamide (10 mg/kg)	1*	80

*P < .05, compared to castor oil group, chi-square test.
